# A protocol for a scoping review of methodologies used to explore patient experience in post-acute rehabilitation settings

**DOI:** 10.12688/hrbopenres.13672.1

**Published:** 2023-01-16

**Authors:** Zsofia Torok, Aisling O’Keeffe, Andrew Darley, Áine Carroll

**Affiliations:** 1 School of Medicine, University College Dublin, Dublin, D04 V1W8, Ireland; 2 National Rehabilitation Hospital, Dublin, A96 E2H2, Ireland

**Keywords:** Patient Experience, Patient Feedback, Healthcare Improvement, Quality of Health Care, Rehabilitation, Methodology, Scoping Review

## Abstract

**Background:** Patient experience is routinely collected in the clinical environment in many different ways throughout various person-provider encounters, but so far limited research focused on understanding the methods of using it to improve the quality of healthcare. This paper presents a protocol for a methodological scoping review examining the methods of obtaining, analysing, reporting, and using patient experience data for quality improvement in rehabilitation settings.

**Methods:** The scoping review will be conducted according to the guidelines from the Joanna Briggs Institute (JBI) Manual for Evidence Synthesis and the methodological framework by Arksey & O’Malley. A comprehensive search of the literature will be performed using a three-step search strategy: an initial limited search of two databases was already performed and helped to identified relevant key words and index terms. The developed search string will be adapted and applied across four databases. This will be followed by search of the reference lists of selected sources and hand-search relevant data-hubs. Studies with a clear focus on patient experience or feedback collected or used for healthcare improvement in rehabilitation context, will be included. A data extraction framework will be developed and piloted to guide the literature screening and data charting. Qualitative content analysis will be employed to address research questions and the results will be presented – beside the descriptive format - as a map of data in chart and tabular formats.

**Conclusions:** This scoping review will show the extent and scope of the literature on the applied methods of collecting, communicating, and using patient experience for quality improvement in post-acute rehabilitation settings and will evaluate and map the evidence on these topics. The findings will inform a research project entitled “An exploration into the use of patient experience to inform improvement in a National Rehabilitation Hospital”.

## Introduction

Patient experience is an important feature of patient-centred care; interest in which has been increasing in recent years. Patient-centred care has been valued as a means to enhance quality of care in healthcare settings ( Richards *et al.*, 2015) The aim of patient-centred care is to effectively address the needs and values of the patient by focusing on including the patient in the decision-making process ( Cheng *et al.*, 2016). The provision of patient-centred care has had a positive impact on patient satisfaction and self-management or self-care practices ( Rathert *et al.*, 2013). Additionally, patient-centred care has been found to be comparable or superior to physician- or disease-centred care ( Morgan & Yoder, 2012; Rathert *et al.*, 2013). In 2015, the World Health Organization published a global strategy on integrated people-centred health services (IPCHS) which highlighted the importance of people- and patient-centred care for improving access to and satisfaction with care, improved health and clinical outcomes, and improved health literacy ( World Health Organization, 2015).

Patient experience encompasses the wide variety of interactions that a patient has within the healthcare system, as well as the patient’s satisfaction with these interactions. Incorporating patient experience into healthcare provision and research has been shown to improve clinical safety and effectiveness ( Doyle *et al.*, 2013), as it allows for the identification of strengths and shortcomings in current services. By understanding patient experience, healthcare systems have the opportunity to implement change and potentially improve services based directly on feedback from the people they intend to serve and avoids prescribing what is assumed to be valuable. There has been a significant increase in research regarding Patient and Public Involvement (PPI) ( Brett *et al.*, 2014). PPI entails working in collaboration or partnership with patients, carers, service users or the public in planning, designing, managing, conducting, dissemination and translation of research ( Stewart *et al.*, 2011). By involving patient experience in the improvement of services, it may be possible to create care experiences that are not only effective but personally meaningful to patients. The collection of patient experience data offers a different, non-clinical perspective on the operation of healthcare systems and is thus an essential consideration in the improvement of healthcare delivery.

Collection of patient experience data is unlikely to solely lead to significant changes in clinical practice ( Ahmed *et al.*, 2014). Though opportunities to give feedback are regularly offered to patients across many health disciplines (for example, the National Patient Experience Survey which commenced in 2017) ( Health Information and Quality Authority, 2017), no change will come into effect unless this feedback is acted upon. It is the responsibility of healthcare providers to incorporate and integrate patient feedback into efforts to improve healthcare practice and service delivery. Failure to do so is unethical; it is morally incorrect to request feedback from patients if that feedback will be ignored ( Coulter *et al.*, 2014). Therefore, it is essential to not only understand how patient experience data is collected but how learnings from this data are integrated to inform change in healthcare settings.

Recent systematic reviews broadly examined this topic in healthcare. Gleeson *et al.* (2016) reviewed approaches to using patient experience data for quality improvement in healthcare settings. They found that patient experience data were most commonly collected by using questionnaires. These were typically administered and analysed by external organisations who offered limited support for healthcare staff to interpret and use the results. The collected patient experience data were typically used to identify minor areas of process changes that did not require major structural alterations or changes to the practice. While most studies reported having made effective improvements, the authors found it difficult to determine the actual changes or ascertain their impact. Cadel *et al.* (2022) reviewed studies that used patient experience data to inform quality improvement and found a wide range of quality improvement initiatives implemented in hospital settings but limited information on the process of using patient experience data and how initiatives were implemented. They concluded that there is a broad understanding in the literature that it is important to act on patient experience data, but less consensus exists on how these data should be actioned.

The authors decided to limit the present review to the field of rehabilitation because it is being undertaken to inform a research project conducted in a rehabilitation setting. However, it can be argued that rehabilitation patients differ from acute care patients in such a way that it is reasonable to focus on them as a separate population. Rehabilitation patients more often live with chronic impairments, co-morbidities, and face more complex problems, in consequence receiving care for a longer period of time and meeting a larger number and variety of healthcare professionals. It is especially important therefore to capture and learn from their perceptions and experiences across various components of received care to ensure the quality of the person-centred care in rehabilitation.

A recently published scoping review examined the use of patient experience feedback for person-centred rehabilitation quality improvement ( Jesus *et al.*, 2022). It focused on the outcomes and included papers that contained an analysis of the impact of the quality improvement informed by patient experience data. They identified only a few examples of the use of patient experience feedback in quality improvement or codesign activities in the rehabilitation literature. They also found that these patient experience improvement activities relied exclusively on retrospective experience survey data, not making use of more actionable, real-time, and inclusive forms of patient experience feedback. The authors of the present review consulted the lead author in the preparation of the current protocol to discuss remaining gaps in the current evidence synthesis. The team made the decision to focus on the process of collection and usage of patient experience feedback. Therefore, the aim of the current scoping review is to examine the methods of obtaining, analysing, reporting, and using patient experience data to inform quality improvement in post-acute rehabilitation settings.

## Methods

A scoping review is described as “ *a type of evidence synthesis that aims to systematically identify and map the breadth of evidence available on a particular topic, field, concept, or issue (…) within or across particular contexts*.” ( Munn *et al.*, 2022)

The protocol for this scoping review was developed under the methodological guidance of Levac *et al.* (2010) and the Joanna Briggs Institute (JBI) Manual for Evidence Synthesis guidelines ( Peters *et al.*, 2020). As outlined in Arksey and O’Malley (2005), the scoping reviews often contain iterative processes, and the researchers potentially may need to revisit previous stages in the light of newly acquired knowledge. These changes to the protocol will be reported in the final paper, which will be composed in accordance with the Preferred reporting Items for Systematic Reviews and Meta-analyses (PRISMA) extension for Scoping Reviews (PRISMA-ScR) ( Tricco *et al.*, 2018).

The methodological framework by Arksey and O’Malley consists of six stages: (1) identifying the research question, (2) identifying relevant studies, (3) selecting studies, (4) charting/mapping the data, (5) collating, summarising, and reporting results and (6) expert consultation. The framework will be guiding this review protocol and the subsequent scoping review.

*The authors received ethical approval for conducting the scoping review from NRH Research Ethics Committee (date of approval 24/05/2022) and the UCD Office of Research Ethics (Research Ethics Exemption Reference Number LS-E-22-06-Carroll, date of approval 13/01/22).*

### Stage 1: Identifying the research question

Following the PRISMA-ScR guidelines, the Population, Concept, Context (PCC) framework was used to guide the framing of the research question and key screening criteria.

- Population: patients, clients, or users of healthcare services
- Concept: the process of collecting, analysing, reporting, and using patient experience, satisfaction, or feedback for the improvement of quality of health care
- Context: studies conducted in rehabilitation healthcare settings: rehabilitation hospitals, centres, clinics, long-term care facilities or out-patient services in any country.

Based on the above, the following research question was identified:

*How are patient experience data collected, analysed, reported and used for healthcare improvement within a rehabilitation setting?*

### Stage 2: Identifying relevant studies

Following the recommendation by the JBI Manual for Evidence Synthesis ( Peters *et al.*, 2020), a three-step process will be used to carry out the search. Step 1 – a preliminary search for relevant articles on two online databases – has already been performed, followed by an analysis of the titles, abstracts, and index terms of the retrieved papers to identify the relevant key terms and phrases. Step 2 will involve the search using the identified key words (as outlined in Table 1) across the included databases. In Step 3, reference lists of selected sources will be manually searched to identify any additional relevant studies. In addition, we will compile a list of measures and instruments widely used for capturing patients’ feedback and perform a search for these applied in rehabilitation settings to find studies relevant to our review.

**Table 1.** Keywords for the search strings.

Patient experience		Healthcare improvement			Rehabilitation setting	Exclude
Patient	Experience	Quality of health care		Improvement	Rehabilitation	
patient*	experience*	healthcare	quality	improv*	rehabilitati*	drug
client*	satisfaction	“health care”	process	enhanc*	physiotherapy	addict*
person*	feedback		performance		“physical therapy”	alcohol*
inpatient*	percep*		procedure		“occupational therapy”	substance
in-patient*	percei*		practice		neurorehabilitation	abuse
outpatient*	perspective*				habilitation	misuse
out-patient*	view*					“mental health”
user	preference*					narcotic*
people	attitude* narrative*					cardiac
						pulmonary
						psychiatr*
						telerehabilitation
						telehealth

A comprehensive search of the literature will be undertaken within the following databases:

- OVID MEDLINE
- CINAHL Nursing and Allied Health (CINAHL Plus)
- APA PsycINFO
- COCHRANE Database of Systematic Reviews

Grey literature search will be also performed in a range of healthcare-related evidence sources and data-hubs including Lenus, greynet.org and Google.

***Key search terms and search strings.*** The key search concepts for this review are ‘Patient experience’, ‘Healthcare improvement’ and ‘Rehabilitation setting’. Keywords, together with synonyms, alternative and related terms will be used to build a search string, and exclusion terms will be added to eliminate results linked to acute medical rehabilitation and substance abuse rehabilitation. Table 1 contains the search terms and exclusion terms for the search strings.

The search query was built from the identified key terms, and adapted for each database using Boolean operators, proximity operators, truncation markers, and applicable MeSH-, subject- and index terms and headings. An expert university librarian was consulted for designing the search strategy. Table 2 contains the search strings for each database.

**Table 2.** Search strings for each database.

Database	Core search (title, abstract)	MeSH-, subject- or index terms
OVID MEDLINE	(patient* or client* or person* or inpatient* or in-patient* or outpatient* or out- patient* or user or people) adj3 (experience* or satisfaction or feedback or percep* or percei* or attitude* or perspective* or view* or preference or narrative*) and (care or healthcare or health care) and (quality or process or performance* or procedure* or practice*) and (improv* or enhanc*) and (rehabilitati* or physiotherapy or physical therapy or occupational therapy or neurorehabilitation or habilitation)	*patient satisfaction/ or Patient Advocacy/ or Patient Reported Outcome Measures/ or Personal Narratives as Topic/ and *"quality of health care"/ or *quality improvement/ or Quality Assurance, Health Care/ or Benchmarking/ or Staff Development/ or Inservice Training/ or Routinely Collected Health Data/ or "Public Reporting of Healthcare Data"/ or Organizational Innovation/ or "Diffusion of Innovation"/ or Organizational Case Studies/ or Models, Organizational/ or Knowledge Management/ or Change Management/ or Capacity Building/ or Clinical Governance/ or Management Information Systems/ or Organizational Culture/ or Program Development/ or Hospital-Patient Relations/ and *rehabilitation/ or *hospitals, rehabilitation/ or *"physical and rehabilitation medicine"/ or *stroke rehabilitation/ or *neurological rehabilitation/ or *rehabilitation centers/ or *rehabilitation research/ or *occupational therapy/ or Rehabilitation Nursing/
CINAHL	(patient* OR client* OR person* OR inpatient* OR in-patient* OR outpatient* OR out-patient* OR User OR People) N3 (experience* OR satisfaction OR feedback OR percep* OR percei* OR attitude* OR perspective* OR view* OR preference OR narrative*) AND (care OR healthcare OR “health care”) AND (quality OR process OR performance* OR procedure* OR practice*) AND (improv* OR enhanc*) AND (rehabilitati* OR physiotherapy OR “physical therapy” OR “occupational therapy” OR neurorehabilitation OR habilitation)	(MH "Patient Satisfaction") OR (MH "Patient Advocacy") AND (MH "Quality of Health Care") OR (MH "Quality Assessment") OR (MH "Quality Improvement") OR (MH "Benchmarking") OR (MH "Quality Assurance") OR (MH "Quality Management, Organizational") OR (MH "Implementation Science") OR (MH "Stakeholder Participation") OR (MH "Organizational Development") OR (MH "Program Development") OR (MH "Quality of Care Research") OR (MH "Organizational Change") AND MH "Activities of Daily Living+") OR (MH "Rehabilitation") OR (MH "Occupational Therapy") OR (MH "Physical Therapy") OR (MH "Rehabilitation, Speech and Language") OR (M "Rehabilitation Centers") OR (MH "Rehabilitation Nursing")
PsycINFO	(patient* OR client* OR person* OR inpatient* OR in-patient* OR outpatient* OR out-patient* OR user OR people) N3 (experience* OR satisfaction OR feedback OR percep* OR percei* OR attitude* OR perspective* OR view* OR preference OR narrative*) AND (care OR healthcare OR “health care”) AND (quality OR performance OR process* OR procedure* OR practice*) AND (improv* OR enhanc*) AND (rehabilitati* OR physiotherapy OR "physical therapy" OR "occupational therapy" OR neurorehabilitation OR habilitation)	MM "Client Satisfaction" AND MM "Quality of Care" OR MM "Quality of Services" OR (MM "Organizational Change" OR MM "Organizational Development") AND MM "Rehabilitation" OR MM "Rehabilitation Centers" OR MM "Activities of Daily Living" OR MM "Occupational Therapy" OR MM "Physical Therapy" OR MM "Speech Therapy"
COCHRANE	(patient* OR client* OR person* OR inpatient* OR in-patient* OR outpatient* OR out-patient* OR user OR people):ti,ab,kw AND (experience* OR satisfaction OR feedback OR percep* OR percei* OR attitude* OR perspective* OR view* OR preference OR narrative*):ti,ab,kw AND (care OR healthcare OR “health care”):ti,ab,kw AND (quality OR performance OR process* OR procedure* OR practice*):ti,ab,kw AND (improv* OR enhanc*):ti,ab,kw AND (Rehabilitati* OR “physical therapy” OR “occupational therapy” OR neurorehabilitation OR physiotherapy OR habilitation):ti,ab,kw	[Patient Satisfaction] OR [Patient Advocacy] AND [Quality of Health Care] OR [Quality Improvement] OR [Benchmarking] OR [Quality Assurance, Health Care] OR [Total Quality Management] OR [Organizational Innovation] OR [Change Management] OR [Program Development] AND [Rehabilitation] OR [Rehabilitation Centers] OR [Hospitals, Rehabilitation] OR [Physical and Rehabilitation Medicine] OR [Rehabilitation of Speech and Language Disorders] OR [Rehabilitation Research] OR [Neurological Rehabilitation] OR [Telerehabilitation] OR [Rehabilitation of Speech and Language Disorders] OR [Stroke Rehabilitation] OR [Neurological Rehabilitation] OR [Rehabilitation Nursing]

***Inclusion/exclusion criteria.*** Following the PRISMA-ScR guidelines, the Population, Concept, Context (PCC) framework was used to guide the framing of the research question and key screening criteria. In line with the methodological guidance from Arksey and O’Malley (2005), the final inclusion and exclusion criteria will be refined based on increasing familiarity with the body of literature and type of data available.

All peer-reviewed and non-peer-reviewed articles and reports published in the English language, with a focus on patient experience or feedback in rehabilitation setting, will be considered for inclusion in the review. Studies with a clear focus on patient experience or feedback collected or used for healthcare improvement, conducted within a rehabilitation setting or with relevance to a rehabilitation context will be included. Articles describing collecting patient feedback or testing survey instruments will not be included unless they also include a specific focus on using the collected data for healthcare improvement. Articles will be excluded if they contain no discussion of using patient experience or user satisfaction for the improvement of quality of healthcare and/or are unrelated to rehabilitation settings.

Careful consideration was given to the timeframe of the search strategy and the authors decided to limit the search to the previous ten years of evidence, in order to keep focus on recent findings and relevant contexts.

### Stage 3: Study selection

Before the start of the screening, the team of reviewers will meet to decide on study inclusion and exclusion criteria. All abstracts will be screened by two reviewers and a third reviewer will resolve any conflict and decide on final inclusion. The articles found from the database searches will be imported into the bibliographic reference management software Endnote. Duplicate articles will be removed. The Covidence systematic review software tool will be used for screening of the retrieved literature. Free tools, such as Rayyan, can also be used as an alternative. The reviewers will meet at the beginning, midpoint, and final stages of each review process. At the first meeting, study inclusion and exclusion criteria will be decided on. The reviewers will independently screen 25 titles/abstracts that were randomly selected for the pilot testing process (in accordance with the JBI Manual for Evidence Synthesis), and then meet to discuss the differences and modify the inclusion criteria, if needed. The screening will start when 75% (or greater) agreement is achieved. All abstracts will be screened by two reviewers and a third reviewer will resolve any conflict and decide on final inclusion.

Potentially relevant papers will be retrieved in full and imported into Covidence systematic review software. The full text article review will be undertaken by the same reviewers using the same method, piloting first the screening process on a sample of randomly selected papers. The full text of selected articles will be assessed against the inclusion criteria by two reviewers. Reasons for exclusion of sources that do not meet the inclusion criteria will be documented and reported in the scoping review. Any disagreement will be resolved by a third reviewer.

### Stage 4: Mapping/charting the data

A provisional data charting table, for extracting relevant data from sources deemed eligible for inclusion, will be developed and piloted by the review team at the protocol stage, in line with the JBI Manual for Evidence Synthesis ( Peters *et al.*, 2020).

The following types of information will be extracted:

- Author(s)
- Year of publication
- Journal information
- Origin/country of origin
- Research design and methodology
- Study setting (hospital / community)
- Population characteristics (sample size, patients & family attributes)
- Definition/description of patient experience
- Type of patient experience measure or feedback used
- Methods of patient experience data collection (including timepoint and by whom is the patient experience data collected)
- How patient experience data is used for healthcare improvement
- Healthcare improvement characteristics (improvement initiatives, description, content, method of delivery, frequency, duration)
- Key findings
- Interpretation of the findings
- Recommendations for future research, policy, or practice

A small sample of publications (n=10) will be randomly selected to pilot the data charting table, then the reviewers will meet to ascertain accuracy and consistency and discuss the discrepancies. It is anticipated that data charting will be an iterative and reflexive process and consequently the data chart will be refined throughout the data extraction stage.

### Stage 5: Collating, summarising and reporting the results

The PRISMA-ScR guidelines ( Tricco *et al.*, 2018) will be used to report the findings and a PRISMA flow diagram will be created to report the details of the screening and selection process. As per JBI guidance, the extracted data will be presented in tabular and/or diagrammatic form in a manner that aligns with the objective of this scoping review. Descriptive statistical analysis will be carried out to demonstrate the general characteristics of the included studies, and a narrative synthesis approach will be utilised to answer the research questions. Deductive thematic analysis will be used to synthesise the study findings and provide information on methods of integrating patient feedback into quality improvement in rehabilitation settings. This will allow identification of areas in which a literature gap exists.

This scoping review is being undertaken to inform a research project entitled “ *An exploration into the use of patient experience to inform improvement in a National Rehabilitation Hospital,*” which will investigate the methods of collection and subsequent implementation of patient experience data in a post-acute rehabilitation setting. The findings of this project will be disseminated to key experts and stakeholders within the National Rehabilitation Hospital (NRH). The review will be submitted for publication in a peer reviewed academic journal, and the results will be presented at conferences.

### Stage 6. Expert consultation

Expert consultation - the optional, but recommended stage of the methodological framework by Arksey and O’Malley ( Daudt *et al.*, 2013) is going to be embedded throughout the whole review process. Professional stakeholders (i.e., health and social care professionals, managers) were consulted to identify priorities and consequently to help guide the research question and design. Throughout the course of the scoping review, expert stakeholders will be engaged in consultation to obtain recommendations for the types of data extracted and the presentation of findings, in order to shape the direction of the scoping review so it will support the research project as closely as possible.

### Study status

Step 1 of the three-step process for applying a search strategy ( Peters *et al.*, 2020) has already been completed. A preliminary search for relevant articles has been performed. The titles, abstracts, and index terms of the retrieved papers were analysed to identify the relevant key terms and phrases. Search queries were built and adapted for each database based on the identified terms. The search queries were tested and are ready to be run.

## Conclusion

This scoping review will address a gap in the current evidence synthesis by exploring the methodologies employed to collect patient experience data and how it is implemented within post-acute rehabilitation settings to improve patient care internationally. The findings from this review, as outlined in the current protocol, will highlight the extent and scope of the literature on the topic of using patient experience to improve healthcare delivery in rehabilitation settings. Additionally, the review will map the research on the applied methods of obtaining, analysing, reporting, and using patient experience for quality improvement, as well as an evaluation of the methods employed. While the impetus for this review is to inform a research project (“An exploration into the use of patient experience to inform improvement in a National Rehabilitation Hospital”), the review may be applied to other clinical settings and offer an opportunity for researchers and healthcare providers to reflect on methods used to gather patient experience data in various clinical settings.

## Data Availability

No data are associated with this article.
