# Supplementary material for: A protocol for a scoping review of methodologies used to explore patient experience in post-acute rehabilitation settings
Source: HRB Open Res. 2024 Feb 26;6:5. Originally published 2023 Jan 16. [Version 2] doi: 10.12688/hrbopenres.13672.2 (PMC11401988; doi:10.12688/hrbopenres.13672.2)
Supplement: Peer review history

# Reviewer report: Reviewer response for version 2

**DOI:** 10.21956/hrbopenres.15213.r43240

**Published:** 2024-11-18

**Authors:** Lauren Cadel

**Affiliations:** 1 Trillium Health Partners Foundation, Mississauga, Ontario, Canada

***Abstract:***

- There is a disconnect between abstract and methods about the guidelines followed (JBI and Arksey and O’Malley vs JBI and Levac). I acknowledge that Levac et al. built on the work of Arksey and O’Malley, but consistency between the abstract and methods is recommended to avoid confusion.

***Methods:***

- “Context: studies conducted in rehabilitation healthcare settings: rehabilitation hospitals, centres, clinics, long-term care facilities or out-patient services in any country.” I am interested by the decision to include long-term care facilities as a rehabilitation facility, as in many countries, this would not be conceptualized as such. Providing a definition of what is meant by rehabilitation healthcare settings would be beneficial.
- “Step 1 – a preliminary search for relevant articles on two online databases – has already been performed, followed by an analysis of the titles, abstracts, and index terms of the retrieved papers to identify the relevant key terms and phrases.” Please specify what databases were searched.
- As per PRISMA-ScR, please provide a rationale for all exclusion criteria.
- Please explain how duplicate articles will be removed.

***Additional Comments:***

 There are a few long, run-on sentences; I suggest the authors review both the introduction and methods to simplify where possible.

Is the study design appropriate for the research question?

Yes

Is the rationale for, and objectives of, the study clearly described?

Partly

Are sufficient details of the methods provided to allow replication by others?

Partly

Are the datasets clearly presented in a useable and accessible format?

Not applicable

Reviewer Expertise:

NA

I confirm that I have read this submission and believe that I have an appropriate level of expertise to confirm that it is of an acceptable scientific standard, however I have significant reservations, as outlined above.

# Reviewer report: Reviewer response for version 2

**DOI:** 10.21956/hrbopenres.15213.r40402

**Published:** 2024-09-15

**Authors:** Deborah Edwards

**Affiliations:** 1 Cardiff University, Cardiff, Wales, UK

JBI no longer refer to themselves as the Joanna Briggs Institute - please just use JBI

 In the abstract please list the databases and the search dates

 In the main text please provide the dates of the search and why the restriction to English language. 

 I would suggest additionally searching SCOPUS of Web of Science.

 Please provide a definitive list of grey literature sites and do you mean an advanced Google. 

 Please provide the details of the databases where the preliminary search was conducted and the full search strategies including the number of hits for each line 

 If you are using Covidence then there is no need to mention Rayyan 

 In the abstract you mention citation tracking but I cannot find this in the manuscript. 

 Evidence synthesis is an overarching term for different types of systematic reviews. There please replace the line "Evidence synthesis will not be performed..." with a more suitable word such as thematic synthesis.

Is the study design appropriate for the research question?

Yes

Is the rationale for, and objectives of, the study clearly described?

Yes

Are sufficient details of the methods provided to allow replication by others?

Partly

Are the datasets clearly presented in a useable and accessible format?

Not applicable

Reviewer Expertise:

Evidence synthesis

I confirm that I have read this submission and believe that I have an appropriate level of expertise to confirm that it is of an acceptable scientific standard, however I have significant reservations, as outlined above.

# Reviewer report: Reviewer response for version 2

**DOI:** 10.21956/hrbopenres.15213.r38331

**Published:** 2024-09-14

**Authors:** Abubeker Alebachew Seid

**Affiliations:** 1 Samara University, Semera, Ethiopia

Manuscript greatly improved than before.

Is the study design appropriate for the research question?

Yes

Is the rationale for, and objectives of, the study clearly described?

Yes

Are sufficient details of the methods provided to allow replication by others?

Yes

Are the datasets clearly presented in a useable and accessible format?

Not applicable

Reviewer Expertise:

Physiotherapy and rehabilitation, Musculoskeletal disorders, Systematic review and meta analysis

I confirm that I have read this submission and believe that I have an appropriate level of expertise to confirm that it is of an acceptable scientific standard.

# Reviewer report: Reviewer response for version 2

**DOI:** 10.21956/hrbopenres.15213.r40397

**Published:** 2024-06-24

**Authors:** Jean-Baptiste GARTNER

**Affiliations:** 1 Université Laval, Québec, Canada

**Comments to the author(s)**

 This study protocol formulates a proposal for a scoping review using the JBI guideline and the Arksey & O'Malley framework.

 INTRODUCTION/LITERATURE REVIEW

 - The background and the context are rather well presented, and the writing is fluid.

 - However, certain points need to be clarified:

 1. The statement "The aim of patient-centred care is to effectively address the needs and values of the patient by focusing on including the patient in the decision-making process” needs to be reformulated. Nowadays, the decision-making process is just one component, not the only one.  For this literature, see: reference 1-3

 2. “Patient experience encompasses the wide variety of interactions that a patient has within the healthcare system, as well as the patient’s satisfaction with these interactions.” (Reference ? ). See reference 4-6

 3. Patient and Public Involvement: See 

- 10.1186/s12961-020-00644-3 (reference 7)
- 10.1016/j.healthpol.2021.07.008 (reference 8)

- Overall: However, I find the literature too limited. In addition, I find it problematic that the literature cited in the introduction does not go beyond 2017. In fact, there is a wealth of literature on these topics and many more recent articles exist. My suggestions for improvements are along these lines.

 I invite you to strengthen your motivation to justify the scoping review and your positioning:

 - A search for protocols and reviews published in recent years should be carried out in the Cochrane Database, the JBI Database of Systematic Reviews and Implementation Reports and PROSPERO to show how your scoping review proposal fits in with existing or ongoing work.

 - Position yourself at least in relation to : 

- 10.1016/j.healthpol.2022.07.008 (reference 4)
- 10.1186/s13012-018-0784-z (reference 6)   

METHODS

 The methodology is interesting and diverse. A minor change could be made:

 - “the Joanna Briggs Institute (JBI) Manual for Evidence Synthesis guidelines (Peters et al., 2020)”. The JBI manual is in its 2024 version : https://jbi-global-wiki.refined.site/space/MANUAL

Some typos and errors:

 Introduction: settings (Richards et al., 2015) . The

 Good luck for the next steps.

Is the study design appropriate for the research question?

Yes

Is the rationale for, and objectives of, the study clearly described?

Partly

Are sufficient details of the methods provided to allow replication by others?

Yes

Are the datasets clearly presented in a useable and accessible format?

Not applicable

Reviewer Expertise:

Health services research

I confirm that I have read this submission and believe that I have an appropriate level of expertise to confirm that it is of an acceptable scientific standard, however I have significant reservations, as outlined above.

## References

[1] : Defining and implementing patient-centered care: An umbrella review. *Patient Educ Couns* .2022;105(7) : 10.1016/j.pec.2021.11.004 1679-1688 10.1016/j.pec.2021.11.004 34848112

[2] : "Same same or different?" A review of reviews of person-centered and patient-centered care. *Patient Educ Couns* .2019;102(1) : 10.1016/j.pec.2018.08.029 3-11 10.1016/j.pec.2018.08.029 30201221

[3] : How can healthcare organizations implement patient-centered care? Examining a large-scale cultural transformation. *BMC Health Serv Res* .2018;18(1) : 10.1186/s12913-018-2949-5 168 10.1186/s12913-018-2949-5 29514631 PMC5842617

[4] : The uses of Patient Reported Experience Measures in health systems: A systematic narrative review. *Health Policy* .2023;128: 10.1016/j.healthpol.2022.07.008 1-10 10.1016/j.healthpol.2022.07.008 35934546

[5] : The Patient Experience: Informing Practice through Identification of Meaningful Communication from the Patient's Perspective. *Healthcare (Basel)* .2018;6(1) : 10.3390/healthcare6010026 10.3390/healthcare6010026 29558392 PMC5872233

[6] : Engaging patients to improve quality of care: a systematic review. *Implement Sci* .2018;13(1) : 10.1186/s13012-018-0784-z 98 10.1186/s13012-018-0784-z 30045735 PMC6060529

[7] : Exploring the theory, barriers and enablers for patient and public involvement across health, social care and patient safety: a systematic review of reviews. *Health Res Policy Syst* .2021;19(1) : 10.1186/s12961-020-00644-3 8 10.1186/s12961-020-00644-3 33472647 PMC7816359

[8] : The impact of patient and public involvement in health research versus healthcare: A scoping review of reviews. *Health Policy* .2021;125(9) : 10.1016/j.healthpol.2021.07.008 1208-1221 10.1016/j.healthpol.2021.07.008 34376328

# Reviewer report: Reviewer response for version 1

**DOI:** 10.21956/hrbopenres.14951.r36013

**Published:** 2023-11-29

**Authors:** Abubeker Alebachew Seid

**Affiliations:** 1 Samara University, Semera, Ethiopia

The study is clearly presented and of scientific quality. Patient experience in post rehabilitation setting is a key component of the rehabilitation service which should be measured and used as an input to improve the service. This scoping review will add new information in this regard.

 In some sections too detailed information (not needed for scoping review) is presented and might induce some confusion such as the use of PCC framework (usually used for systematic review of observational studies) and too much quantitative data extraction and or analysis.

**Scoping review is** implemented to map the literature on a certain topic and to identify gaps. It will be better if authors consider this in stating the aim of the study.

 The current protocol looks as a full “systematic review” protocol rather than scoping review protocol. It needs to be contextualized for a scoping review protocol, mainly methods section and “Stage 5: Collating, summarising and reporting the results” section.

  

1. The study focus on “rehabilitation patients more often live with chronic impairments, co-morbidities, and face more complex problems, in consequence receiving care for a longer period of time……” and the title mentioned about **patient experience in post-acute rehabilitation settings. While the treatment is taking longer time and involves multidisciplinary professionals why authors want to focus on “post-acute rehabilitation”? or What is “post-acute rehabilitation” refers in this study?**
2. Regarding the “Population, Concept, Context (PCC) framework”; the framework might narrow your search results. Your research question is again wider than the above-mentioned framework. The research question has an OUTCOME assessment. It is not clear why authors are choosing the PCC framework for a scoping review.   
3. As per the protocol (see also mapping/charting the data) you have data for both qualitative and quantitative analysis. Authors should have to consider this (probably not going to much on the quantitative data extraction/analysis) to align with their research design (Scoping review)

Is the study design appropriate for the research question?

Yes

Is the rationale for, and objectives of, the study clearly described?

Yes

Are sufficient details of the methods provided to allow replication by others?

Yes

Are the datasets clearly presented in a useable and accessible format?

Not applicable

Reviewer Expertise:

Physiotherapy and rehabilitation, Musculoskeletal disorders, Systematic review and meta analysis

I confirm that I have read this submission and believe that I have an appropriate level of expertise to confirm that it is of an acceptable scientific standard, however I have significant reservations, as outlined above.

# Response

**Published:** 2024-02-16

**Authors:** Zsofia Torok

**Affiliations:** UCD School of Medicine, Ireland

***The study is clearly presented and of scientific quality. Patient experience in post rehabilitation setting is a key component of the rehabilitation service which should be measured and used as an input to improve the service. This scoping review will add new information in this regard.***

***In some sections too detailed information (not needed for scoping review) is presented and might induce some confusion such as the use of PCC framework (usually used for systematic review of observational studies) and too much quantitative data extraction and or analysis.***

***Scoping review is implemented to map the literature on a certain topic and to identify gaps. It will be better if authors consider this in stating the aim of the study.***

***The current protocol looks as a full “systematic review” protocol rather than scoping review protocol. It needs to be contextualized for a scoping review protocol, mainly methods section and “Stage 5: Collating, summarising and reporting the results” section.***

**Response:**

 Thank you for the feedback and drawing our attention to omissions.

 The development of the review protocol was guided by the Chapter 11: Scoping reviews in JBI Manual for Evidence Synthesis (Peters et al., 2020) that recommends using the PCC to guide the review objective and the inclusion criteria.

 In order to clearly state the objective of mapping the literature and identifying gaps, we are proposing to complete the aim of the study section with listing the following objectives:

 Objectives:

1. To explore and categorize the diverse methodologies employed for collecting patient experience data in post-acute rehabilitation settings, assess their implementation, and investigate their impact on improving patient care internationally.
2. To determine the extent and scope of existing literature related to the applied methods of collecting, communicating, and utilizing patient experience data for quality improvement in post-acute rehabilitation settings.
3. To identify and highlight gaps in the current literature pertaining to the methods used for collecting patient experience data in post-acute rehabilitation settings.

We are also proposing a revision for the Stage 5: Collating, summarising and reporting the results section, located on page 12.

***1.         The study focus on “rehabilitation patients more often live with chronic impairments, co-morbidities, and face more complex problems, in consequence receiving care for a longer period of time……” and the title mentioned about patient experience in post-acute rehabilitation settings. While the treatment is taking longer time and involves multidisciplinary professionals why authors want to focus on “post-acute rehabilitation”? or What is “post-acute rehabilitation” refers in this study?***

**Response:**

 Thank you for drawing our attention to this omission.

 In the present study, 'post-acute rehabilitation' refers to the phase of care that occurs after the acute phase, involving ongoing rehabilitation efforts to optimize functional outcomes. In our opinion, post-acute rehabilitation represents a critical phase in the continuum of care after the acute phase. Understanding the methods of obtaining and utilizing patient experience data in this specific phase is essential for comprehensively addressing the diverse and long-term needs of individuals in their recovery journey.

 As patients transition from acute care to post-acute rehabilitation, the emphasis often shifts towards patient-centered care, as acute care primarily focuses on stabilizing and treating immediate medical issues, allowing less emphasis on individualized and holistic patient preferences.

 Another reason for focusing on post-acute rehabilitation is that the review's findings will inform a research project within a post-acute rehabilitation hospital. Therefore, we wanted the patient population of the review to resemble the hospital’s patient profile, which does not include patients in their acute stages of their injury. This rationale is reflected in the revised fifth paragraph in the Introduction section on page 4. and quoted here:

*"The authors decided to limit the present review to the field of rehabilitation because it is being undertaken to inform a research project conducted in a post-acute rehabilitation hospital. Specifically focusing on post-acute rehabilitation patients within this context allows for a targeted exploration of their unique experiences and needs. Rehabilitation patients more often live with chronic impairments, co-morbidities, and face more complex problems, in consequence receiving care for a longer period of time and meeting a larger number and variety of healthcare professionals. Additionally, present study examines post-acute rehabilitation as a crucial phase in patient care after the acute stage. It is especially important therefore to capture and learn from the perceptions and experiences across various components of received care to ensure the quality of the person-centred care in rehabilitation."*

***2.         Regarding the “Population, Concept, Context (PCC) framework”; the framework might narrow your search results. Your research question is again wider than the above-mentioned framework. The research question has an OUTCOME assessment. It is not clear why authors are choosing the PCC framework for a scoping review.***

**Response:**

 Thank you for the feedback.

 The development of the review protocol was guided by the Chapter 11: Scoping reviews in JBI Manual for Evidence Synthesis (Peters et al., 2020) that recommends using the PCC to guide the review objective and the inclusion criteria. Our initial scoping search of the literature showed that patient experience has been studied using heterogenous methods. We chose to use the PCC framework to ensure that the search strategy captured both qualitative and quantitative research with may have be inhibited using the PICOT framework which is generally used for quantitative studies.

***3.         As per the protocol (see also mapping/charting the data) you have data for both qualitative and quantitative analysis. Authors should have to consider this (probably not going to much on the quantitative data extraction/analysis) to align with their research design (Scoping review)***

**Response:**

 Thank you for the feedback and the advice.

 For quantitative data analysis, we are planning to conduct simple frequency counts, to recognize patterns and trends, and use visual representations to compare findings across studies, with a focus on identifying themes and potential research gaps.

 For the qualitative data, we are aiming to perform a descriptive qualitative content analysis, employing basic coding and categorization methods to analyse and label extracted data, and identify recurring themes and patterns.

 This is reflected in the revision of Stage 5 description, located on page 12.

# Reviewer report: Reviewer response for version 1

**DOI:** 10.21956/hrbopenres.14951.r35270

**Published:** 2023-07-26

**Authors:** Bronwyn Simpson

**Affiliations:** 1 The University of Sydney, Sydney, New South Wales, Australia

The purpose of the protocol (and subsequent study) are clear and understanding methods of collecting patient experiences will make an important contribution to the literature.

 A scoping review is a suitable design for the study aims.

 The methods are appropriate and clearly described. The use of deductive thematic analysis (under stage 5) is a little confusing and needs elaboration - will a framework be used for this deductive analysis? The research questions? Some inductive analysis also seems appropriate here.

 Quality appraisal is not mentioned - the mapping of the research should ideally include some kind of appraisal and reporting on methodological quality of the included studies.

Is the study design appropriate for the research question?

Yes

Is the rationale for, and objectives of, the study clearly described?

Yes

Are sufficient details of the methods provided to allow replication by others?

Yes

Are the datasets clearly presented in a useable and accessible format?

Yes

Reviewer Expertise:

Qualitative research, scoping reviews, case study research in the field of disability-related inclusion and well-being.

I confirm that I have read this submission and believe that I have an appropriate level of expertise to confirm that it is of an acceptable scientific standard, however I have significant reservations, as outlined above.

# Response

**Published:** 2024-02-16

**Authors:** Zsofia Torok

**Affiliations:** UCD School of Medicine, Ireland

***The use of deductive thematic analysis (under stage 5) is a little confusing and needs elaboration - will a framework be used for this deductive analysis? The research questions? Some inductive analysis also seems appropriate here.***

**Response:** Thank you for the feedback. After careful consideration we decided against a full thematic analysis, and decided to employ basic coding and categorization methods that are more appropriate for a scoping review in order to identify trends and relevant comparison and/or divergence. This is reflected in the revision of Stage 5 description, located on page 12.

***Quality appraisal is not mentioned - the mapping of the research should ideally include some kind of appraisal and reporting on methodological quality of the included studies.***

**Response: **Thank you for drawing our attention to this omission.

 According to the Chapter 11: Scoping reviews in JBI Manual for Evidence Synthesis (Peters et al., 2020), the formal assessment of methodological quality of the included sources of evidence is not required for scoping reviews. We are planning to conduct an inclusive and comprehensive mapping of literature, aiming for a broader overview of the topic without conducting formal assessments of the methodological quality of included studies. Our approach will involve transparent reporting, narrative synthesis, and the identification of research gaps to inform future investigations, acknowledging the diverse range of evidence.

 To make it clear for the protocol, we are proposing to complete the penultimate paragraph in the section *Stage 2: Identifying relevant studies* with a following sentence:

*As formal assessment of methodological quality of the included studies is not required for scoping reviews, appraisal of the methodological quality will not be performed.*
